# Hydride Accessibility and Reactivity in the Configurational and Stoichiometric Space of β‑Ga_2_O_3_ for CO_2_ Hydrogenation

**DOI:** 10.1021/acs.jpclett.5c01571

**Published:** 2025-07-24

**Authors:** Margareth S. Baidun, Alexander A. Kolganov, Anastassia N. Alexandrova, Evgeny A. Pidko

**Affiliations:** † Inorganic Systems Engineering, Department of Chemical Engineering, Faculty of Applied Sciences, 2860 Delft University of Technology, Van der Maasweg 9, 2629 HZ Delft, The Netherlands; ‡ Department of Chemistry and Biochemistry, 8783 University of California, Los Angeles, California 90094, United States

## Abstract

Understanding how surface species evolve under reaction conditions is essential for improving catalyst design for efficient CO_2_ hydrogenation. This work combines systematic DFT calculations with grand canonical sampling to investigate the stability and reactivity of Ga–H species on β-Ga_2_O_3_ across a range of reaction conditions. Initial DFT studies reveal that when Ga–H species are present, they facilitate formate formation via a low-barrier pathway, largely independent of the surface termination or hydrogen site. However, grand canonical sampling shows that under a broad range of reaction conditions—especially at high oxygen chemical potentials associated with high water content—Ga–H species are thermodynamically inaccessible. Furthermore, adsorbed water molecules can block reactive sites, inhibiting CO_2_ activation even when hydrides are present. These findings suggest that the lack of accessible hydride species, rather than their intrinsic reactivity, could contribute to reduced catalytic performance of β-Ga_2_O_3_ under more oxidizing, high-conversion conditions.

## Graphical abstract

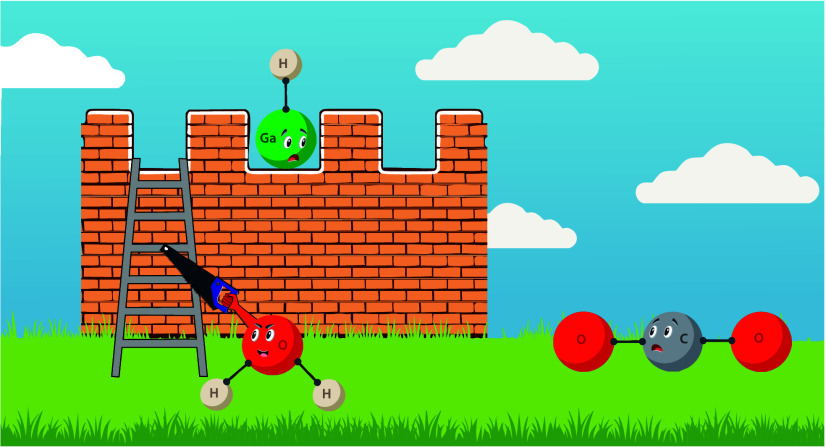

Understanding the structure and reactivity of catalyst surfaces is crucial for rational design, requiring detailed insights into processes such as adsorption/desorption and reaction pathways. Density functional theory (DFT) has been instrumental in advancing our understanding of these processes in heterogeneous catalysis, offering valuable atomistic-scale insights. However, the static nature of many computational studies, their focus on local minima structures, and reliance on preselected surface models often overlook the complex, dynamical effects that arise from ensemble behavior under realistic catalytic conditions.^1–3^ These missing factors are critical in catalytic systems where surface composition, coverage, and restructuring dictate reactivity.^4–6^ In contrast, ensemble-based approaches offer a broader thermodynamic perspective, capturing variations in oxidation states, adsorbates, and surface stoichiometry under various conditions, while allowing for detailed site-specific insights.

In this work, detailed systematic DFT calculations and grand canonical sampling are combined to provide a comprehensive perspective on CO_2_ hydrogenation over gallium oxide (β-Ga_2_O_3_). This oxide has emerged as a promising catalytic material, with demonstrated promotional effect in bimetallic systems.^7–13^ In addition, Ga_2_O_3_ has been extensively studied as a standalone material in reactions such as propane dehydrogenation,^14,15^ the water–gas shift reaction,^16^ and CO_2_ hydrogenation.^17^ Recent studies have identified surface Ga–H species and proposed their potential relevance in facilitating CO_2_ activation.^18^ Similar hydride intermediates have also been detected on other group 13 oxides, such as In_2_O_3_ and Al_2_O_3_, where they have been proposed to play a key role in CO_2_ hydrogenation.^19–21^ Given their potential importance in CO_2_ activation, determining whether Ga–H species are thermodynamically stable and prevalent under realistic reaction conditions is essential.

To address this question, we first conducted a systematic DFT study of various β-Ga_2_O_3_ surfaces, identifying the stability and reactivity of the hydrides across different terminations (steps (1) and (2) in Figure 1). These detailed findings were incorporated into an ensemble-based approach where the configurational and stoichiometric space of β-Ga_2_O_3_ was mapped through grand canonical sampling across a range of reaction conditions (step (3)). This ensemble-based approach allowed us to identify relevant surface hydride species and assess their reactivity under realistic conditions (step (4)), providing both detailed mechanistic insights and a broader thermodynamic understanding.

**Figure 1:**
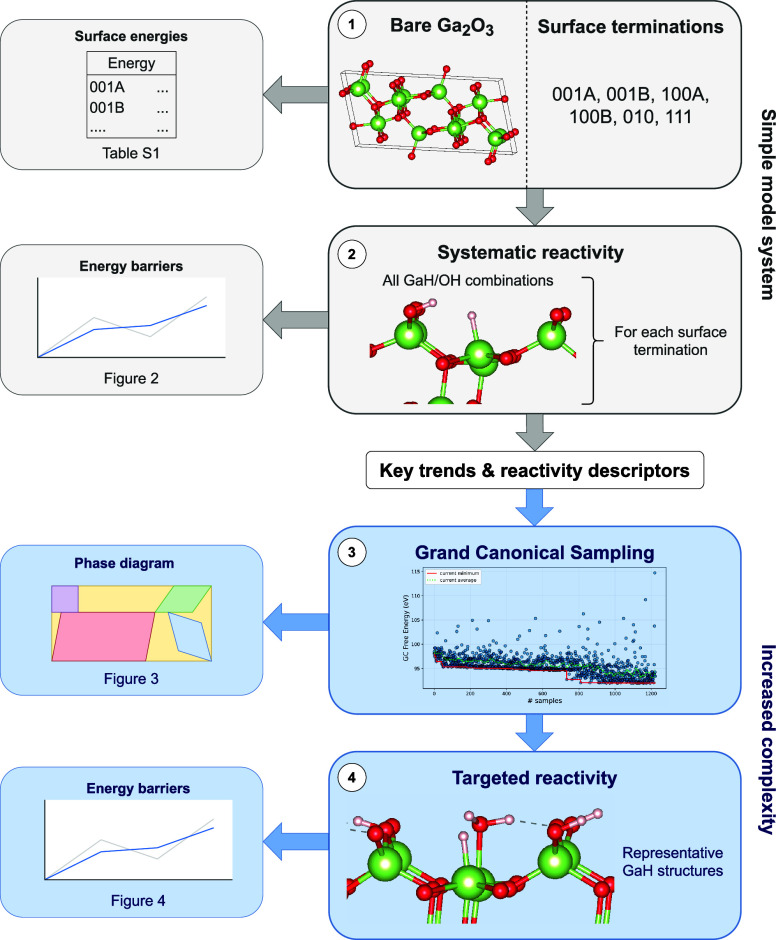
Summary of the computational workflow. Starting from a simple model of bare β-Ga_2_O_3_, six surface terminations were considered (1). All possible GaH/OH combinations resulting from heterolytic H_2_ splitting were evaluated for each surface, and their reactivity toward CO_2_ was assessed (2). Insights from this model system formed a hypothesis, which was tested using grand canonical sampling to generate a more realistic surface ensemble (3). Representative hydride structures from the ensemble were then subjected to targeted reactivity calculations (4).

All DFT calculations were performed using VASP^22–25^ with the PBE functional^26^ and DFT-D3­(BJ) dispersion corrections.^27^ Geometry optimizations in steps (1), (2), and (4) employed a 550 eV plane wave cutoff energy, spin polarization, and a 1 × 2 × 1Monckhorst-Pack *k*-point grid to sample the Brilluon zone. Structures were relaxed until forces on all unconstrained atoms were below 0.04 eV/Å, with electronic convergence set to 10^–5^ eV. For the high-throughput sampling in step (3), a more efficient setup was used, with a reduced cutoff of 400 eV, a single Γ-point, and a slightly relaxed force threshold of 0.06 eV/Å. Further computational details are provided in the Supporting Information.

We started by systematically exploring the structural characteristics of β-Ga_2_O_3_ and the stability of its surface terminations. The monoclinic unit cell consists of two inequivalent Ga sites—octahedral *Ga­(1)* and tetrahedral *Ga­(2)*—and three inequivalent O sites: 3-fold-coordinated *O­(1)*, 3-fold-coordinated *O­(2)*, and tetrahedral *O­(3)*. These inequivalent sites lead to multiple possible cleavage terminations for low-index surfaces, labeled as ”A” and ”B”. Six surface terminations were examined: 100A, 100B, 001A, 001B, 010, and 111, exposing different Ga and O sites. The calculated surface energies (see Supporting Information S2) follow the trend in literature values,^28^ with the 100B surface as the most stable.

Although the exact dominant mechanism for H_2_ activation on Ga_2_O_3_ remains challenging to confirm experimentally, several studies indicate that Ga–H formation through heterolytic H_2_ splitting is likely to occur, resulting in Ga–H and O–H species in close proximity.^18,29–31^ To explore the role of these hydrides in CO_2_ activation, we systematically studied H_2_ dissociation on each surface termination, considering all possible combinations of Ga and O sites. The reactivity of Ga–H species was assessed by their reaction with CO_2_, with particular focus on formate (HCOO) formation, a key intermediate in methanol synthesis.^18,30^ Although some mechanistic studies show that methanol formation through the COOH intermediate is possible,^10,11,32^ the HCOO pathway is widely considered the dominant route for methanol formation.^18,29,33–35^ In related systems such as In_2_O_3_, the HCOO pathway is favored over the COOH route, which proceeds via CO formation and can reduce methanol selectivity.^36^

A summary of the systematic reactivity study across different surfaces is shown in Figure 2. For each surface, a representative pathway is shown, with full details and alternative paths provided in the Supporting Information. Across all studied surfaces, if formate is formed, its formation proceeds via surface Ga–H species.^18,36^ Despite significant differences in surface stability, as indicated by the relative positions of the surface energy levels in the figure, the computed energy barriers for formate formation are similar across the surfaces, ranging from 0.33 to 0.56 eV. These barriers suggest that if hydrides are present, reactivity is likely to be comparable across the surfaces, regardless of their relative stability.

**Figure 2:**
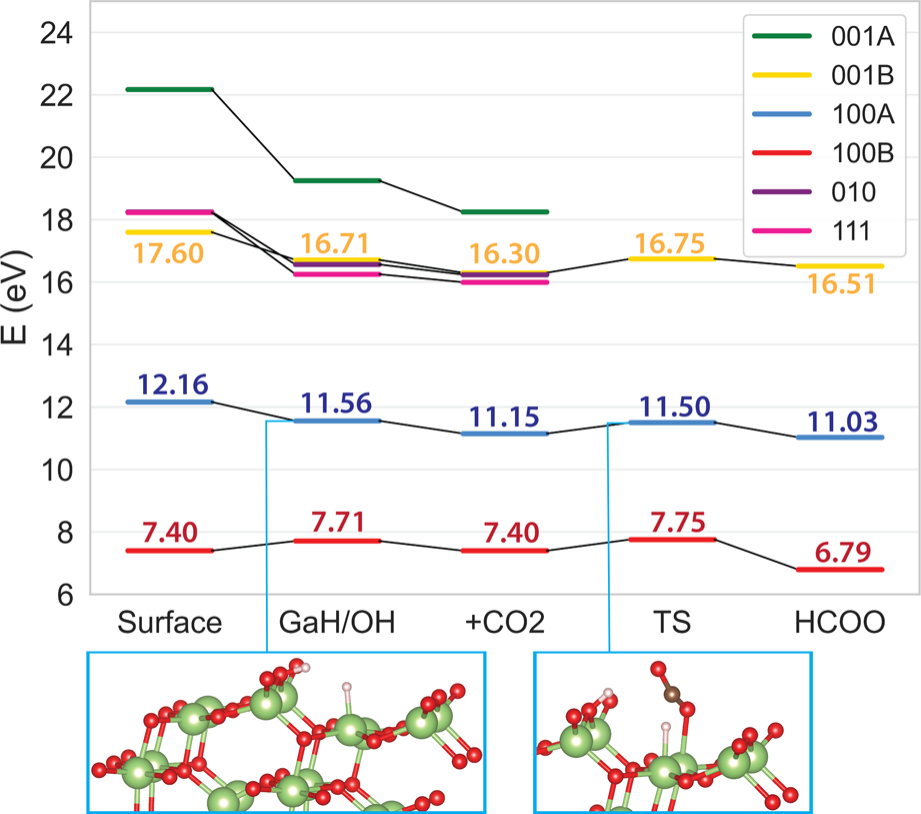
Pathways for the first steps of CO_2_ hydrogenation to formate on different surface terminations of β-Ga_2_O_3_. From each termination, a representative pathway is shown. All energies are referenced to the corresponding number of β-Ga_2_O_3_ bulk units.

Additionally, the most stable surface is not necessarily the most reactive. While the 100B surface has the lowest surface energy, all H_2_ dissociation pathways on this surface are endothermic, indicating lower reactivity (see Supporting Information S3). In contrast, the 010 and 111 surfaces exhibit highly exothermic H_2_ splitting and HCOO formation (Supporting Information S3) but may suffer from surface poisoning under catalytic conditions, inhibiting further methanol formation. The 100A and 001B surfaces each exhibit at least one exothermic H_2_ dissociation pathway with moderately stable HCOO species, making them more promising for reactivity. Between these two, 100A was selected as a representative case for further study, as it offers both a favorable reactivity profile and a moderate surface energy. In addition, this surface has been shown to be experimentally accessible based on a detailed surface energy analysis of Ga_2_O_3_ terminations that accounts for macroscopic facet formation.^37^

These results suggest that when surface Ga–H species are present, their reactivity depends weakly on the precise surface composition or structure, within the limits of the studied models. Given these insights, the key question becomes whether these hydride species are present under realistic catalytic conditions and whether relevant hydride structures exhibit the same expected reactivity, according to the formulated hypothesis. To answer these questions, we extended our investigation using a grand canonical genetic algorithm (GCGA) as implemented in the GOCIA Python package.^38,39^ This ensemble-based method allows us to go beyond idealized surfaces with a single adsorbate by systematically sampling a wide configurational and stoichiometric space, including variations in hydrogen coverage and oxidation states of Ga.

Building on our DFT insights, we focused on the 100A surface and employed GCGA to generate a realistic ensemble of surface structures under varying hydrogen and oxygen chemical potentials. In the sampling procedure, only hydrogen and oxygen atoms were allowed to vary, resulting in a wide range of surface stoichiometries, including Ga–H bonds, O–H bonds, and water molecules. Carbon-containing species were not explicitly included to limit the chemical space and separate the effect of the environment in stabilizing hydrides. Reactivity toward CO_2_ was instead assessed separately using targeted reaction pathway calculations (*vide infra*). Further computational details regarding the sampling are provided in the Supporting Information. This approach enabled us to construct a phase diagram that captures the stability of surface hydrides under different reaction conditions, helping to assess both thermodynamic stability and reactivity of catalytically relevant hydride structures.

The resulting phase diagram, shown in Figure 3(a), illustrates surface composition across a wide range of reaction conditions. The structure used in the initial DFT calculations — a perfect surface slab with a single Ga–H/O–H pair (Ga_32_O_48_H_2_) — does not correspond to any global minimum on the phase diagram, emphasizing the importance of grand canonical sampling to obtain realistic surface representation. Interestingly, the operating conditions used in the experiment (indicated by the green star) lie near a phase boundary between Ga_32_O_52_H_8_ and Ga_32_O_52_H_9_. This observation aligns with recent studies across a range of catalytic systems, where activity often correlates with phase boundaries.^40^ When moving away from this boundary, we expect activity to decline. At the same time, it is notable that none of the global minimum structures across the phase diagram contain Ga–H bonds. Figure 3(b) quantifies the energy difference between the global minimum and the first Ga–H containing phase. Under the chosen reaction conditions (indicated by the green star), hydrides are thermodynamically unfavorable. However, under more reducing conditions—particularly at lower oxygen chemical potentials—Ga–H species become accessible within 0.5 eV, suggesting that these hydride-containing phases could be stabilized under suitably tuned environments.

**Figure 3:**
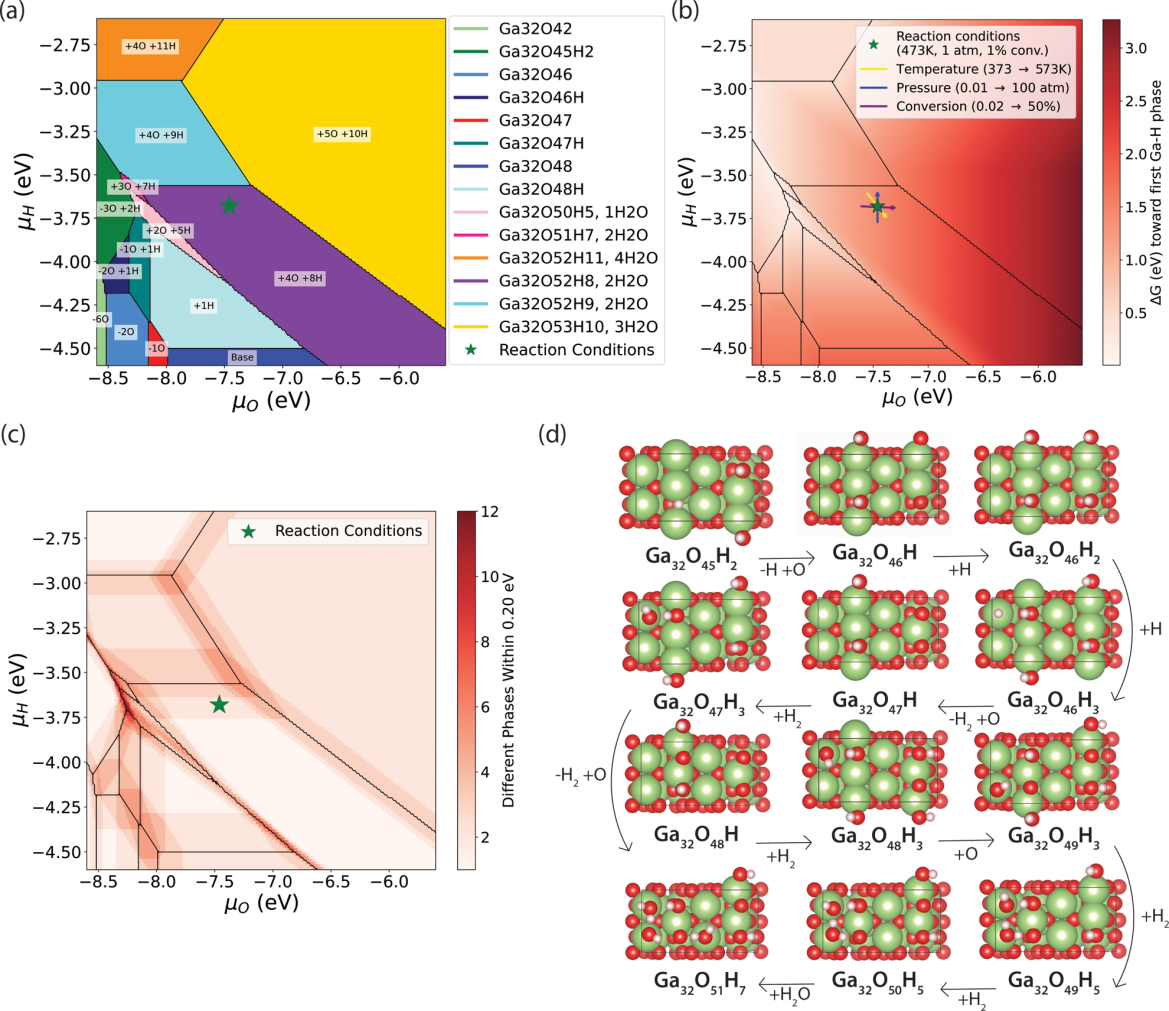
(a) Phase diagram of β-Ga_2_O_3_(100A) under various reaction conditions. The green star denotes the chemical potentials for the chosen reaction conditions (200 °C, 1 atm, 1:3 CO_2_:H_2_, assumed 1% conversion). When hydrogen is not part of a water molecule, it forms O–H bonds. (b) Energy from global minimum to first Ga–H-containing phase. Arrows indicate the change of the position of the reaction conditions with change in CO_2_ conversion (purple, change from 0.02 to 50%), pressure (blue, change from 0.01 to 100 atm), and temperature (yellow, change from 373 to 573 K). At the reaction conditions, hydrides are 1.41 eV above the global minimum. (c) Number of distinct phases within an energy range of 0.20 eV from the global minimum. (d) Top view of 12 distinct phases within 0.20 eV at μ_*O*_ = −8.25 eV and μ_*H*_ = −3.71 eV.

We next consider the practical implications of tuning the environment. As the arrows in Figure 3(b) indicate, the chemical potentials of the catalyst environment evolve with temperature, pressure, and CO_2_ conversion. Lower conversion correlates with reduced water content and hence lower μ_O_, which shifts the system toward the region of the phase diagram where Ga–H species are more readily stabilized. This shift suggests that β-Ga_2_O_3_ is most catalytically active under low-conversion conditions, where hydrides are thermodynamically accessible. To estimate how zero-point energy (ZPE) effects might alter this picture, we applied ZPE corrections to a subset of representative structures. These corrections lowered the energy gap toward Ga–H phases by approximately 0.2 eV (see Supporting Information for details). Based on this value, we examined how many distinct surface structures lie within 0.2 eV of the global minimum, shown in Figure 3(c). At μ_*O*_ = −8.25 eV and μ_*H*_ = −3.71 eV, for example, up to 12 surface stoichiometries—ranging from Ga_32_O_45_H_2_ to Ga_32_O_51_H_7_—are found within this range.

This analysis highlights what impact small energetic shifts have, such as those introduced by ZPE corrections. Nonetheless, the main conclusion still holds, that hydrides are not thermodynamically accessible under reaction conditions but become more accessible as water content decreases. This shift toward stable hydrides at lower μ_O_ also moves the system into a region of increased structural flexibility (Figure 3(c) and (d)). This diversity indicates substantial structural adaptability, where transitions between reduced and oxidized states can readily occur. In contrast, under high-conversion conditions—downstream in the reactor—higher water content leads to increased μ_O_, pushing the system into a more rigid region of the phase diagram. Here, Ga–H species become thermodynamically inaccessible and only two phases coexist within the same energy range. These findings are consistent with previous reports of limited hydride formation and low CO_2_ hydrogenation activity on β-Ga_2_O_3_.^41^ Combining the findings on hydride inaccessibility and low barriers for HCOO, we propose that it is the lack of accessible Ga–H species under catalytic conditions that reduces reactivity.

To test this hypothesis, we assessed the reactivity of GCGA-generated hydrides, as shown in Figure 4(a). The selected set consists of five representative hydride configurations: four structures accessible under reducing conditions (hydrides 1–4), and the most stable hydride at the chosen reaction conditions (hydride 5). Notably, two of these structures (3 and 5) contain adsorbed water molecules. In situation 5, this adsorbed water blocks reactive sites, preventing the formation of formate. We removed the water molecules and reoptimized the structures to evaluate how the stability and reactivity of the hydrides are affected, with the results shown in Figure 4(b). Upon water removal, structure 5 becomes less thermodynamically stable. However, both cases now facilitate formate formation through a low-barrier, hydride-mediated transition state. The calculated energy barriers for all identified transition states remain below 0.50 eV, consistent with our findings from the initial systematic DFT studies. These results reinforce our hypothesis: when Ga–H species are present and adjacent sites are not covered with water, CO_2_ activation proceeds readily.

**Figure 4:**
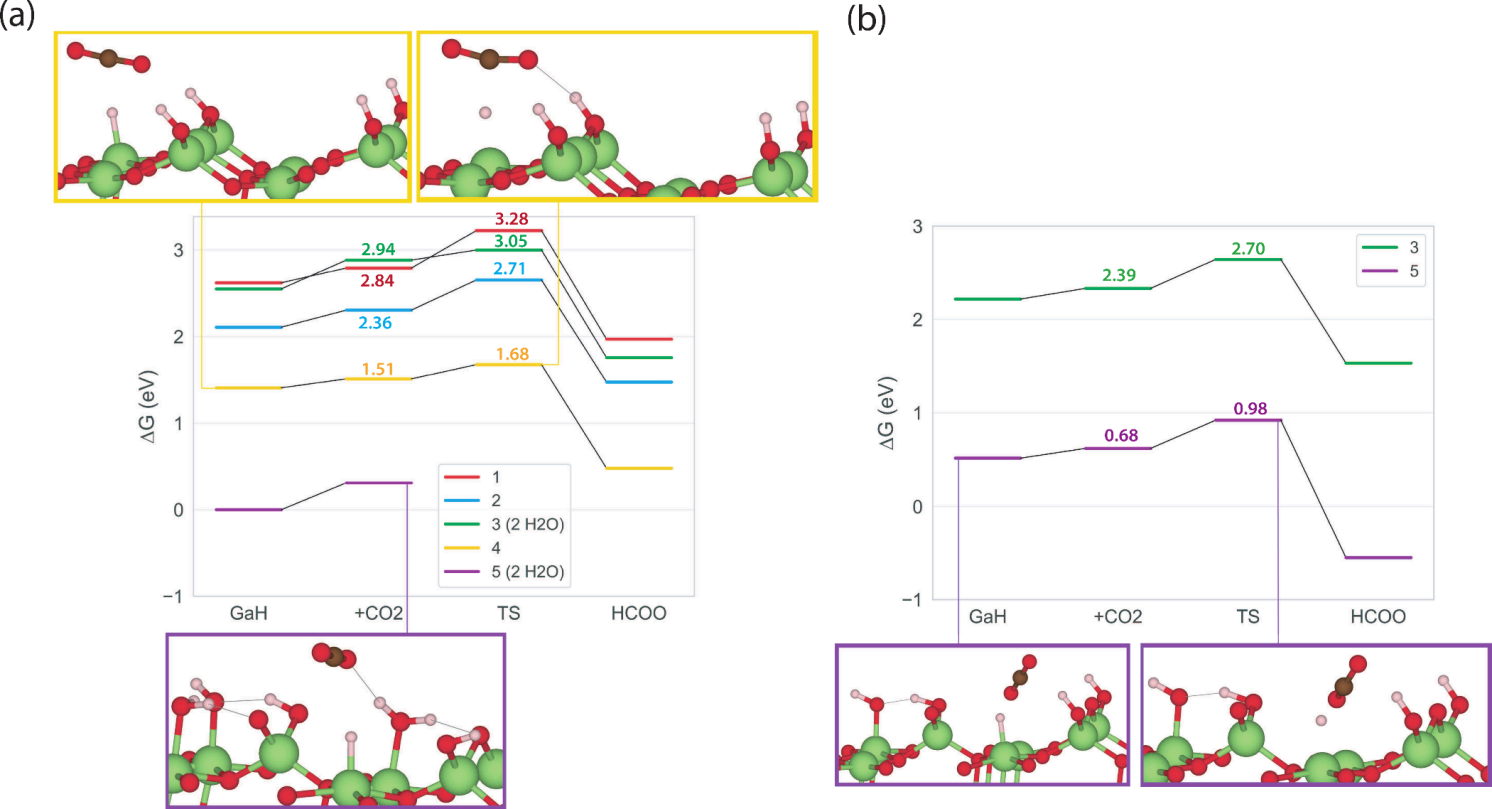
(a) Reactivity of a selection of hydrides as obtained by GOCIA and (b) with water molecules removed if applicable. Structures 4 and 5 are shown as example illustrations. The lowest hydride-containing structure at reaction conditions (structure 5) is chosen as reference.

This study offers a new perspective on the role of surface hydrides on Ga_2_O_3_. By combining systematic DFT calculations with grand canonical ensemble sampling, we propose that once Ga–H species are present, the reaction step toward HCOO proceeds readily. However, the phase diagram reveals that under a broad range of reaction conditions, hydrides are not thermodynamically stable - particularly at high oxygen chemical potentials - suggesting that β-Ga_2_O_3_ may exhibit limited catalytic activity under high conversion conditions. Moreover, environments with high water content not only destabilize surface hydrides but also inhibit HCOO formation by occupying adjacent reactive sites. While the detailed grand canonical approach was performed on the 100A surface, the intrinsic reactivity trends observed across multiple β-Ga_2_O_3_ terminations suggest similar hydride reactivity once hydrides are present. Thermodynamic hydride stability, however, may vary across surfaces and could be explored in future work. The phase diagrams may provide guidance for selecting experimental reaction conditions that enhance hydride availability, helping to optimize CO_2_ hydrogenation. Experimental validation under these conditions can create a positive feedback loop, refining computational models for more accurate predictions. Additionally, stabilizing hydrides remains a key objective.

## Supplementary Material



## Data Availability

All inputs and outputs for the DFT calculations, data sets, and code for data processing are available together with an extensive README via 4TU.ResearchData (10.4121/4d682c30-a979-46e1-bbe3-793ce725ac3c).

## Notes

Computational setup, DFT details, grand canonical sampling details, surface energies ,reaction pathways, zero-point energy corrections, and structure stability (PDF)
