# Supplementary material for: Hydride Accessibility and Reactivity in the Configurational and Stoichiometric Space of β‑Ga2O3 for CO2 Hydrogenation
Source: J Phys Chem Lett. 2025 Jul 24;16(30):7732–7. doi: 10.1021/acs.jpclett.5c01571 (PMC12319897; doi:10.1021/acs.jpclett.5c01571)
Supplement: Supplementary file 1 [file jz5c01571_si_001.pdf]

Supporting Information

Hydride Accessibility and Reactivity in the Configurational and Stoichiometric Space of β-Ga_2O_3 for CO_2 Hydrogenation

Margareth S. Baidun ,[†] Alexander A. Kolganov ,[†] Anastassia N. Alexandrova ,[‡] and Evgeny A. Pidko [*,†]

†*Inorganic Systems Engineering, Department of Chemical Engineering, Faculty of Applied Sciences, Delft University of Technology, Van der Maasweg 9, 2629 HZ, Delft, The Netherlands*

‡*Department of Chemistry and Biochemistry, University of California, Los Angeles, California 90094, United States*

E-mail: e.a.pidko@tudelft.nl

S1 Computational details

S1.1 System set-up

The bulk of monoclinic β - Ga_2O_3 was modeled with a unit cell containing four Ga_2O_3 units, taken from ref 1. After full cell optimization, the obtained lattice parameters were a = 12.439 Å, b = 3.086 Å, and c = 5.890 Å, in good agreement with experimental values (a = 12.214 Å, b = 3.037 Å, and c = 5.798 Å[1]). Six different surface terminations were chosen - 001A, 001B, 100A, 100B, 010, and 111 - with lateral cell dimensions approximating 12

x 6 Å^2 and a minimum of six layers, depending on the termination. The bottom half of each slab was fixed at its bulk positions, while the top half was fully relaxed. A vacuum layer of 12 Å was added in the z-direction to avoid spurious interactions between periodic images. For the GOCIA calculations, the 100A surface termination of Ga_2O_3 was used with the slab thickness reduced to four layers for computational efficiency, while maintaining a fixed bottom layer.

S1.2 DFT calculations

All DFT calculations were performed using the Vienna ab initio simulation package (VASP)[2–5] version 5.4.4, employing the projector-augmented wave (PAW) pseudopotentials[6] with the PBE functional[7] and the DFT-D3(BJ) method for dispersion corrections.[8] A cut-off energy of 550 eV and a 1×2×1 k-point grid (generated via the Monkhorst–Pack scheme) were used for all slab calculations, ensuring energy convergence. Structure relaxations were carried out using either the conjugate gradient or the quasi-Newton algorithms until the forces on all unconstrained atoms were smaller than 0.04 eV/Å. The convergence criterium for electronic optimization was set to 10^{-5} eV and spin-polarization was included in all calculations. Transition state searches were performed using the nudged elastic band (NEB) method, and all identified transition states were verified to have a single imaginary vibrational frequency.

For the optimization of GOCIA-generated structures, a single Γ-point was used in the reciprocal space due to the large number of sampled structures. The energy cut-off was set to 400 eV and the convergence criterium for force minimization was changed to 0.06 eV/Å, in line with previous GOCIA studies.[9]

S1.2.1 Targeted reaction profiles

A subset of representative hydride-containing structures was selected for targeted reactivity analysis. this set includes the most stable Ga-H-containing phase at reaction conditions (structure 5) as well as four additional hydride structures (structures 1–4) that are most

thermodynamically accessible under reducing conditions. The goal is to assess the reactivity of a diverse set of Ga–H structures at the chosen reaction conditions.

For this subset, the same VASP settings have been applied as in the initial reaction profiles (550 eV energy cutoff, 1x2x1 k-point grid, 0.04 eV/Å force criterium, 10^{-5} eV electronic convergence), followed by normal mode analysis using finite displacement method (δ=0.02 Å) as implemented in VASP. Grand potential energies were obtained through Equation 2 (*vide infra*), with the resulting values further corrected by Gibbs free energy contributions:

$$\Delta G = \Delta H - T\Delta S. \tag{1}$$

For the surface slabs, only vibrational contributions and finite temperature correction to entropy and enthalpy were considered. For the gas-phase CO_2 molecule, also translational and rotational contributions were accounted for within the ideal gas approximation at the pressure of 0.25 atm (corresponding to 1:3 CO_2:H_2 at 1 atm total pressure) and temperature of 473K.[10]

S1.3 Grand Canonical sampling

Grand canonical structure sampling was conducted using the Grand Canonical Genetic Algorithm (GCGA) as implemented in the GOCIA Python package.[11,12] The chosen reaction conditions were T = 473K, atmospheric pressure, and a 1:3 ratio CO_2:H_2, corresponding to conditions used in experimental studies on Ni_5Ga_3 in ref 13. The sampling target was to minimize the grand canonical free energy, Ω, defined as:

$$\Omega = E^{\text{slab}} - E^{\text{bare}} - n_{\text{H}} \cdot \mu_{\text{H}} - n_{\text{O}} \cdot \mu_{\text{O}}, \tag{2}$$

where E^{slab} and E^{bare} represent the electronic energies of the generated and pristine 100A surface structures, respectively. The chemical potentials of H and O were computed as:

$$\mu_{\mathrm{H}} = \frac{1}{2}\mu_{\mathrm{H}_2} \tag{3}$$
$$= \frac{1}{2}\left(E^{gas}_{\mathrm{H}_2} - \Delta\mu_{\mathrm{H}_2}(T, p_{\mathrm{H}_2})\right) \tag{4}$$

and

$$\mu_{\mathrm{O}} = \mu_{\mathrm{H}_2\mathrm{O}} - \mu_{\mathrm{H}_2} \tag{5}$$
$$= E^{gas}_{\mathrm{H}_2\mathrm{O}} - \Delta\mu_{\mathrm{H}_2\mathrm{O}}(T, p_{\mathrm{H}_2\mathrm{O}}) - \left(E^{gas}_{\mathrm{H}_2} - \Delta\mu_{\mathrm{H}_2}(T, p_{\mathrm{H}_2})\right). \tag{6}$$

Here, $E^{gas}_{\mathrm{H}_2}$ and $E^{gas}_{\mathrm{H}_2\mathrm{O}}$ denote the electronic energies of isolated hydrogen and water molecules, respectively. The temperature- and pressure-dependent corrections $\Delta\mu_{\mathrm{H}_2}(T, p_{\mathrm{H}_2})$ and $\Delta\mu_{\mathrm{H}_2}(T, p_{\mathrm{H}_2\mathrm{O}})$ were obtained by interpolating tabulated experimental values.[14] A 1% conversion assumption was made to estimate the partial pressure of water. By allowing O and H atoms to be exchanged with the reservoir during sampling, the GCGA procedure explores a variety of surface stoichiometries, including sub-stoichiometric configurations in which surface oxygen atoms have been removed.

Multiple studies have demonstrated the importance of oxygen vacancies on Ga_2O_3.[15–18] To increase the likelihood of generating oxygen-deficient structures withing the GCGA framework, we applied a correction of 0.34 eV to the oxygen chemical potential. This value corresponds to the energy cost of forming an oxygen vacancy on β-Ga_2O_3, as reported by Pan et al.[16] Without this adjustment, vacancy-containing structures might be under-sampled due to the cost of removing an oxygen atom. With this correction, the final values of the H and O chemical potential become μ_H = -3.68 eV and μ_O = -7.46 eV under the defined reaction conditions.

We emphasize that the exact chemical potentials corresponding to experimental reaction

conditions are not straightforward to define. To address this uncertainty, the grand canonical sampling was performed at three different chemical potential sets: 1) the potentials denoting the chosen reaction conditions as defined above, 2) μ_H = -3.18 eV and μ_O = -7.96 eV, and 3) μ_H = -4.18 eV and μ_O = -6.96 eV. For each set, global and local minima were obtained through the sampling mechanism. All sampled structures were optimized using the DFT settings described above. The final dataset included 2806 structures of which 2581 were considered unique, corresponding to an oversampling rate of 8.72%. Grand canonical free energies were re-evaluated across intermediate values of μ_{O} and μ_{H} using Equation 2. This approach enables sampling of a diverse ensemble of surface configurations, including catalytically relevant defect sites and partially reduced surfaces.

S2 Surface energies

All surface slab models used in this study were constructed as symmetric slabs to eliminate artificial dipole moments. To ensure consistency, all slabs were constructed with comparable surface areas ($\tilde{1}2$ x 6 Å) following surface areas from previous DFT studies on Ga_2O_3 surfaces.[15,16,19] Table S1 presents the calculated surface energies of the selected Ga_2O_3 terminations, along with literature values. The surface energies were computed as follows:

$$\sigma = \frac{1}{2A}\left(E_{\mathrm{slab}} - NE_{\mathrm{bulk}}\right), \tag{7}$$

where A is the surface area (assuming both surfaces are equivalent), E_{slab} is the electronic energy of the slab, E_{bulk} is the electronic energy per bulk unit cell, and N is the number of bulk unit cells contained in the slab.

Table S1: Calculated surface energies (σ, J/m^2) of the relaxed surfaces compared to calculated values.

Surface termination	Calculated σ	Literature σ [20]
001A	2.32	...
001B	1.84	1.40
100A	1.34	1.13
100B	0.82	0.68
010	2.06	2.03
111	1.72	...

S3 Reaction pathways

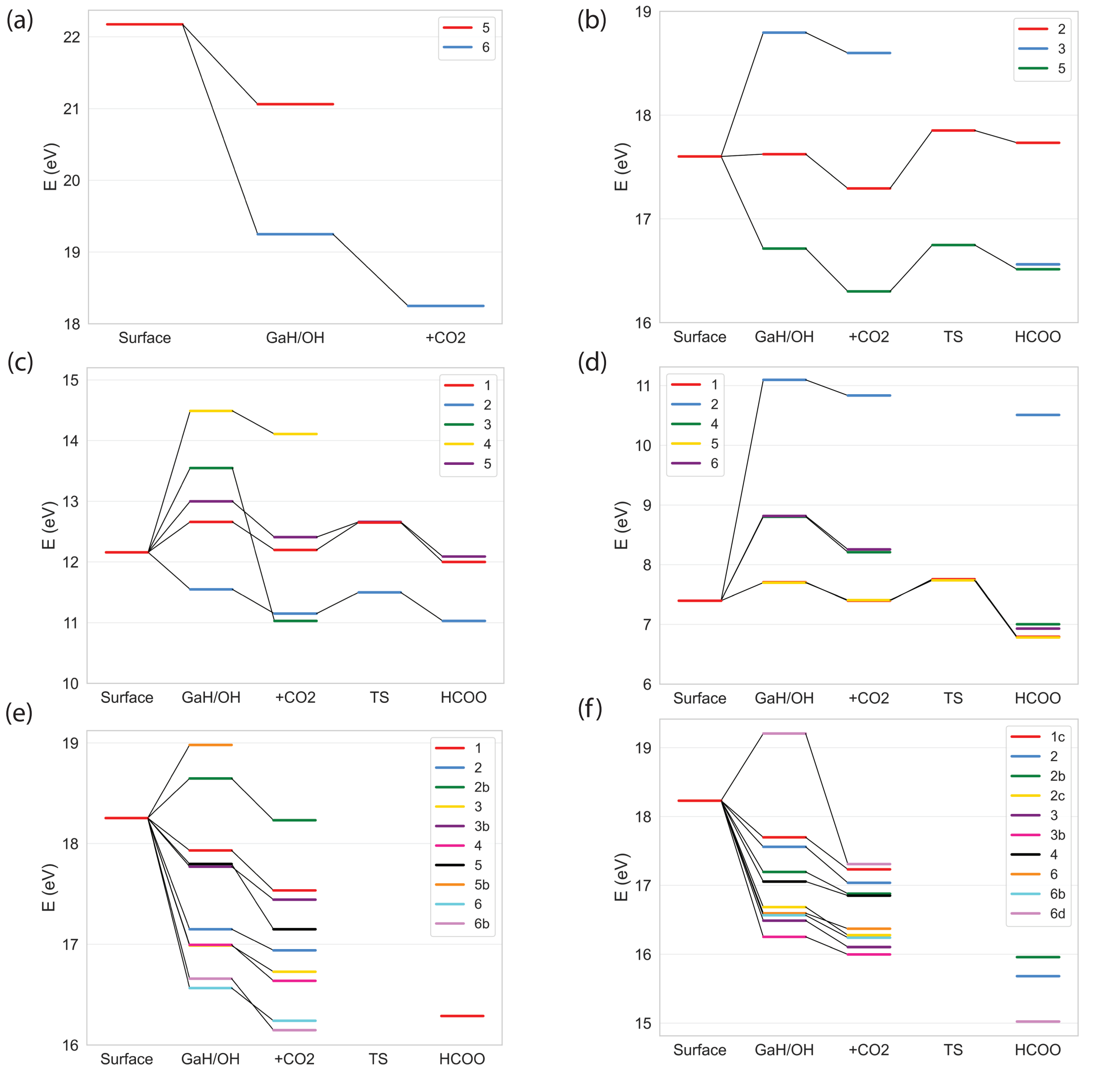

Figure S1: Pathways for the initial steps of CO_2 hydrogenation to formate on various surfaces of β-Ga_2O_3; a) 001A, b) 001B, c) 100A, d) 100B, e) 010, and f) 111. The numbers correspond to the OH/GaH combinations listed in Tables S2 - S7.

S3.1 001A surface

The 001A surface exposes singly undercoordinated *Ga(1)* and *O(2)* sites, while all others maintain bulk coordination. Of the six possible Ga/O combinations for heterolytic H_2 dis-

sociation, only two yielded converged structures. As shown in Figure S1a, both pathways are exothermic relative to the bare 001A surface. Notably, combination 5 involves fully coordinated sites, while combination 6 involves both Ga and O sites that are singly under-coordinated (see Table S2). While dissociation was achieved, no subsequent reaction steps were observed for either pathway. For combination 6, significant surface reconstruction was observed during both H adsorption and CO_2 physisorption (see Figure S2b–c). Upon removal of the adsorbates and re-optimization (Figure S2d–e), the surface relaxed into new configurations that were more stable than the original by 0.64 eV (after H adsorption) and 2.17 eV (after CO_2 adsorption). This degree of stabilization following adsorption aligns with previous findings.[19] However, despite the increased thermodynamic stability, the system showed no further reactivity toward formate formation.

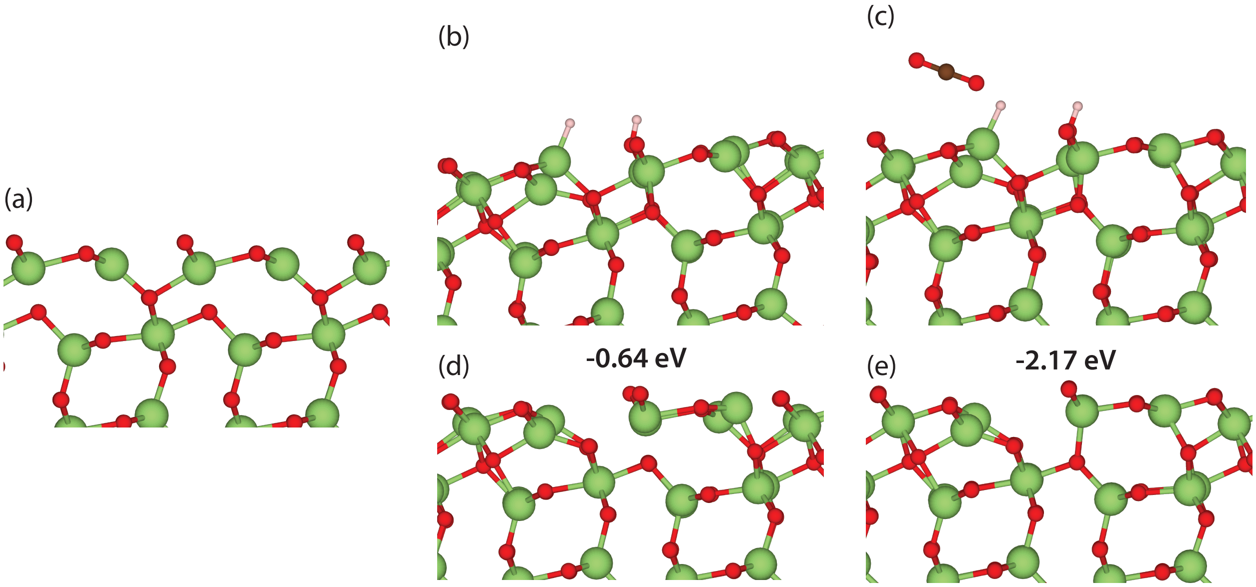

Figure S2: (a) The bare, optimized 100A surface. Surface reconstruction following (b) H-adsorption and (c) CO_2 physisorption. The re-optimized bare surfaces following (d) H-adsorption and (e) CO_2 physisorption, with the given surface stabilities relative to the original surface.

S3.2 001B surface

The 001B cleavage produces doubly undercoordinated *Ga(2)*and *O(3)* atoms. Three OH/GaH combinations have resulted in converged geometries, as shown in Figure S1. Similar to the 001A surface, one of these pathways (combination 3) involves fully coordinated Ga and O atoms (see Table S3). Two of the three pathways progressed to transition states for formate formation, with energy barriers of 0.55 eV and 0.45 eV. These transition states showed bending of the CO_2 molecule and approach of the carbon atom toward the hydride, indicative of hydride-mediated activation. While some surface reconstruction occurred, the surface reverted to its initial configuration upon re-optimization.

S3.3 100A surface

The 100 surface can be cleaved in two distinct ways. The 100A termination exposes singly undercoordinated *Ga(2)* and *O(2)* atoms. Five of six OH/GaH combinations yielded stable structures, one of which was exothermic. Upon CO_2 addition, combination 3 (see Table S4) stood out with an energy stabilization of 1.52 eV, caused by hydride migration to a nearby *O(2)* site, resulting in Ga reduction. This combination did not yield a stable formate. For the other four pathways, stable HCOO* species formed, with three identified transition states. The energy barriers and nature of transition states are comparable to those observed on 001B.

S3.4 100B surface

Cleaving the 100B surface results in singly undercoordinated *Ga(2)* and *O(3)* sites. As the most stable surface (Table S1), all five identified H_2 splitting pathways were endothermic. All five pathways led to stable formate structures, with two identified transition states. Interestingly, combinations 1 and 5, as well as 4 and 6, showed very similar energy profiles despite involving different oxygen sites.

S3.5 010 surface

On the 010 surface, multiple undercoordinated sites are exposed: *Ga(1)*singly, *Ga(2)* doubly, and *O(1)*, *O(2)*, and *O(3)* singly. Due to the cleavage geometry, deeper fully coordinated oxygen atoms ("B" sites) are also accessible and included in OH/GaH pairings. Out of the 12 possible combinations, eight yielded stable OH/GaH structures. Interestingly, although 2b, 3b, 4b, 5b, and 6b involve fully coordinated B-sites (see Table S6), these options produced stable OH/GaH structures, of which 3b and 5b were exothermic. Strong surface reconstruction occurred in the most exothermic pathways. However, unlike 001A, removing adsorbates and re-optimizing the surface did not lead to enhanced stability. Only one stable HCOO structure was found, but no transition state could be identified.

S3.6 111 surface

The cleavage of the 111 surface also exposes subsurface (B) sites, producing a total of 24 OH/GaH combinations. Top-layer oxygen atoms are all singly undercoordinated, while B oxygens remain fully coordinated. Among Ga sites, all *Ga(2)* sites (including B sites) are singly undercoordinated, and *Ga(1)* is singly undercoordinated as well (see Table S7). Ten of the 24 combintations converged, with all but one pathway being exothermic upon H_2 splitting. However, only three stable HCOO* structures were found, and no transition states could be identified.

Table S2: 001A GaH/OH combinations

	Combination	Ga	O
1	Ga(2) - O(1)	-	1
2	Ga(2) - O(2)	-	-
3	Ga(1) - O(2)	1	-
4	Ga(1) - O(3)	1	-
5	Ga(2) - O(3)	-	-
6	Ga(1) - O(1)	1	1

Table S3: 001B GaH/OH combinations

	Combination	Ga	O
1	Ga(2) - O(1)	2	-
2	Ga(2) - O(2)	2	-
3	Ga(1) - O(2)	-	-
4	Ga(1) - O(3)	-	2
5	Ga(2) - O(3)	2	2
6	Ga(1) - O(1)	-	-

Table S4: 100A GaH/OH combinations

	Combination	Ga	O
1	Ga(2) - O(1)	1	-
2	Ga(2) - O(2)	1	1
3	Ga(1) - O(2)	-	1
4	Ga(1) - O(3)	-	-
5	Ga(2) - O(3)	1	-
6	Ga(1) - O(1)	-	-

Table S5: 100B GaH/OH combinations

	Combination	Ga	O
1	Ga(2) - O(1)	1	-
2	Ga(2) - O(2)	1	-
3	Ga(1) - O(2)	-	-
4	Ga(1) - O(3)	-	1
5	Ga(2) - O(3)	1	1
6	Ga(1) - O(1)	-	-

Table S6: 010 GaH/OH combinations

	Combination	Ga	O
1	Ga(2) - O(1)	2	1
1B	Ga(2) - O(1)B	2	-
2	Ga(2) - O(2)	2	1
2B	Ga(2) - O(2)B	2	-
3	Ga(1) - O(2)	1	1
3B	Ga(1) - O(2)B	1	-
4	Ga(1) - O(3)	1	1
4B	Ga(1) - O(3)B	1	-
5	Ga(2) - O(3)	2	1
5B	Ga(2) - O(3)B	2	-
6	Ga(1) - O(1)	1	1
6B	Ga(1) - O(1)B	1	-

Table S7: 100B GaH/OH combinations

	Combination	Ga	O
1	Ga(2) - O(1)	1	1
1B	Ga(2) - O(1)B	1	-
1C	Ga(2)B - O(1)	1	1
1D	Ga(2)B - O(1)B	1	-
2	Ga(2) - O(2)	1	1
2B	Ga(2) - O(2)B	1	-
2C	Ga(2)B - O(2)	1	1
2D	Ga(2)B - O(2)B	1	-
3	Ga(1) - O(2)	1	1
3B	Ga(1) - O(2)B	1	-
3C	Ga(1)B - O(2)	-	1
3D	Ga(1)B - O(2)B	-	-
4	Ga(1) - O(3)	1	1
4B	Ga(1) - O(3)B	1	-
4C	Ga(1)B - O(3)	-	1
4D	Ga(1) - O(3)B	-	-
5	Ga(2) - O(3)	1	1
5B	Ga(2) - O(3)B	1	-
5C	Ga(2)B - O(3)	1	1
5D	Ga(2)B - O(3)B	1	-
6	Ga(1) - O(1)	1	1
6B	Ga(1) - O(1)B	1	-
6C	Ga(1)B - O(1)	-	1
6D	Ga(1)B - O(1)B	-	-

S4 Zero-point energy corrections

Table S8: ΔG toward the first Ga-H phase at three specific points, without (ΔG) and with ZPE correction (ΔG_{ZPE}).

μ_O;μ_H (eV)	ΔG (eV)	ΔG_{ZPE} (eV)
-7.46; -3.68	1.41	1.21
-7.11; -3.49	1.73	1.60
-7.46; -3.49	1.39	1.22

The impact of ZPE corrections was assessed on three μ_O;μ_H points: the chosen reaction conditions (-7.46; -3.68) and the two phases in near proximity to the chosen reaction conditions (-7.11; -3.49) and (-7.46; -3.49). At each point, normal mode analysis was performed for the global minimum and the lowest Ga-H-containing phase. The energy gap toward the lowest Ga-H-containing phase, with and without ZPE corrections, can be found in Table S8.

S5 Structure stability

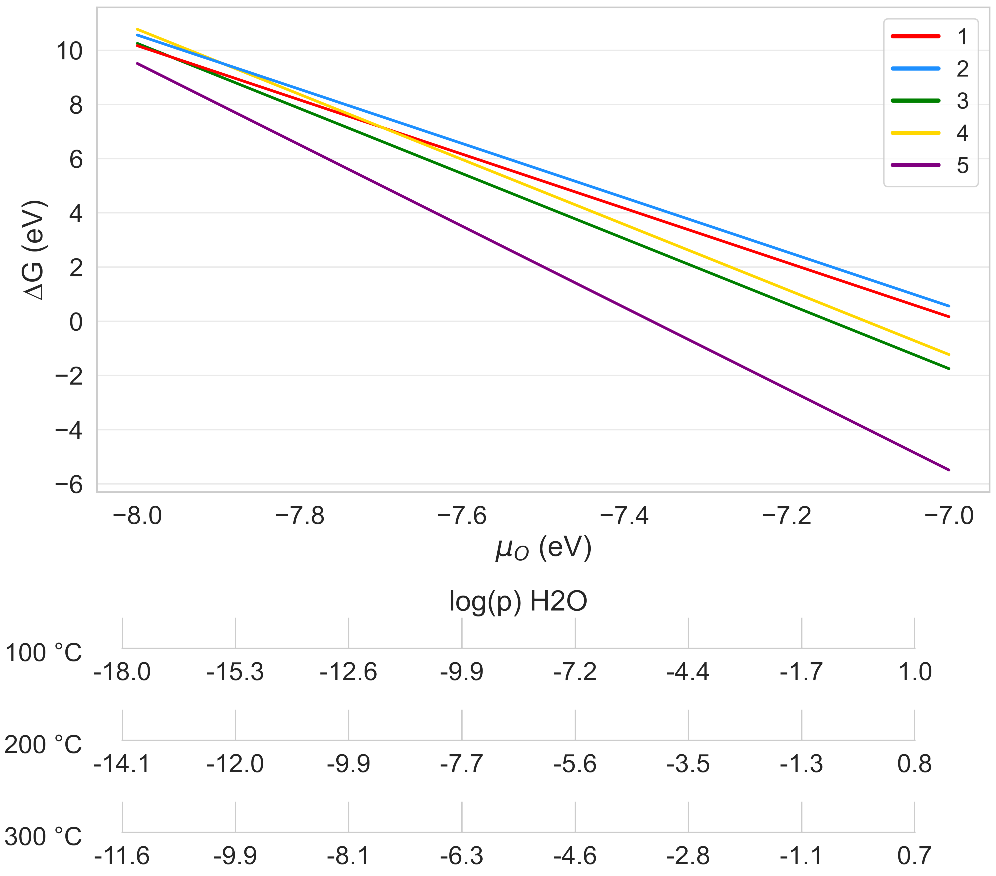

Figure S3: Gibbs free energies for a selection of hydride-containing structures as a function of the oxygen chemical potential, relative to the global minimum at the chosen reaction conditions. Values of μ_O are scaled into the partial pressure of water at different temperatures.

References

(1) Åhman, J.; Svensson, G.; Albertsson, J. A Reinvestigation of ß-Gallium Oxide. *Acta Crystallogr. C* **1996**, *52*, 1336–1338.

(2) Kresse, G.; Hafner, J. *Ab initio* molecular dynamics for liquid metals. *Phys. Rev. B* **1993**, *47*, 558–561.

(3) Kresse, G.; Hafner, J. *Ab initio* molecular-dynamics simulation of the liquid-metal–amorphous-semiconductor transition in germanium. *Phys. Rev. B* **1994**, *49*, 14251–14269.

(4) Kresse, G.; Furthmüller, J. Efficiency of ab-initio total energy calculations for metals and semiconductors using a plane-wave basis set. *Comput. Mater. Sci.* **1996**, *6*, 15–50.

(5) Kresse, G.; Furthmüller, J. Efficient iterative schemes for *ab initio* total-energy calculations using a plane-wave basis set. *Phys. Rev. B* **1996**, *54*, 11169–11186.

(6) Kresse, G.; Joubert, D. From ultrasoft pseudopotentials to the projector augmented-wave method. *Phys. Rev. B* **1999**, *59*, 1758–1775.

(7) Perdew, J. P.; Burke, K.; Ernzerhof, M. Generalized Gradient Approximation Made Simple. *Phys. Rev. Lett.* **1996**, *77*, 3865–3868.

(8) Grimme, S.; Ehrlich, S.; Goerigk, L. Effect of the damping function in dispersion corrected density functional theory. *J. Comput. Chem.* **2011**, *32*, 1456–1465.

(9) Lavroff, R. H.; Cummings, E.; Sawant, K.; Zhang, Z.; Sautet, P.; Alexandrova, A. N. Cu-Supported ZnO under Conditions of CO_2 Reduction to Methanol: Why 0.2 ML Coverage? *J. Phys. Chem. Lett.* **2024**, *15*, 11745–11752.

(10) Ochterski, J. W. Thermochemistry in Gaussian.

(11) Zhang, Z.; Gee, W.; Lavroff, R. H.; Alexandrova, A. N. GOCIA: a grand canonical global optimizer for clusters, interfaces, and adsorbates. *Phys. Chem. Chem. Phys.* **2025**, *27*, 696–706.

(12) Zhang, Z. GOCIA: Global Optimizer for Clusters, Interfaces, and Adsorbates. `https://github.com/zishengz/gocia`, Accessed: March 17, 2025.

(13) Studt, F.; Sharafutdinov, I.; Abild-Pedersen, F.; Elkjær, C. F.; Hummelshøj, J. S.; Dahl, S.; Chorkendorff, I.; Nørskov, J. K. Discovery of a Ni-Ga catalyst for carbon dioxide reduction to methanol. *Nat. Chem.* **2014**, *6*, 320–324.

(14) Allison, T. C. NIST-JANAF Thermochemical Tables - SRD 13. 2013; `https://janaf.nist.gov/`, Accessed: March 17, 2025.

(15) Pan, Y.-x.; Liu, C.-j.; Mei, D.; Ge, Q. Effects of Hydration and Oxygen Vacancy on CO $_2$ Adsorption and Activation on ß-Ga $_2$ O $_3$ (100). *Langmuir* **2010**, *26*, 5551–5558.

(16) Pan, Y.-x.; Mei, D.; Liu, C.-j.; Ge, Q. Hydrogen Adsorption on Ga $_2$ O $_3$ Surface: A Combined Experimental and Computational Study. *J. Phys. Chem. C* **2011**, *115*, 10140–10146.

(17) Gonzalez, E. A.; Jasen, P. V.; Juan, A.; Collins, S. E.; Baltanás, M. A.; Bonivardi, A. L. Hydrogen adsorption on ß-Ga2O3(100) surface containing oxygen vacancies. *Surf. Sci.* **2005**, *575*, 171–180.

(18) Bao, Z.; Jiang, M.; Sun, Z.; Zhang, M.; Dong, J.; Lv, T.; Sun, C.; Chen, X.; Huang, Z.; Yin, P. Manipulating Oxygen Vacancies in $\gamma - Ga_2$ O_3 Nanocrystals: Correlation between Defect Location, Charge State, and Photophysical Properties. *J. Phys. Chem. Lett* **2024**, 12477–12484.

(19) Qu, J.; Tsang, S. C. E.; Gong, X.-Q. A DFT study on surface dependence of ß-Ga2O3 for CO2 hydrogenation to CH3OH. *J. Mol. Model.* **2014**, *20*, 2543.

(20) Bermudez, V. The structure of low-index surfaces of ß-Ga2O3. *Chem. Phys.* **2006**, *323*, 193–203.
